# Advances in Cancer Therapy from Research to Clinical Practice—Surgical, Molecular or Systemic Management of Cancer

**DOI:** 10.3390/medicina59071309

**Published:** 2023-07-14

**Authors:** Calin Cainap, Nicolae Crisan

**Affiliations:** 1 Department of Medical Oncology, Iuliu Hatieganu University of Medicine and Pharmacy, 400012 Cluj-Napoca, Romania; 2 Department of Surgical Specialities, Iuliu Hatieganu University of Medicine and Pharmacy, 400012 Cluj-Napoca, Romania; drnicolaecrisan@gmail.com

Cancer represents one of the most important general health problems of our day. The estimated incidence of new cases in 2020 was 19.3 million [1] versus 10.9 million in 2002 [2], which is an increase of 77%. The future is not very optimistic, as the number of new cases is expected to increase by at least 47% by 2040, reaching 28.4 million new cases [2]. The projections in the US are that, as of January 2022, the total number of patients who were diagnosed with cancer (curable or not, and alive at a definite point of analysis) represented 5.4% of the population [3].

Even when diagnosed at an early stage, cancer patients can experience a metastatic relapse. Gallicchio et al. estimated that, during a lifetime, the percentage of metastasisation can range from 30% for lung cancer to 72% for bladder cancer [4], even though the chance of survival increased throughout the analysed interval of time due to the increasing availability of new categories of drugs used in oncological treatments.

All of these improvements could not be reached without the translation of fundamental research into practical uses, ranging from the initial cellular level to the molecular level, and nowadays, we have a genetic understanding of cancerogenesis. Since the times of ancient Greece, multiple theories have been made about oncogenesis, and through ‘step by step’ discoveries, we have managed to learn how things work in the complex area of human biology, and new potentially valuable targets have emerged.

One of the most important dreams of healthcare personnel—from research, clinical, or laboratory specialities—is to solve a little or big part of the complicated oncology puzzle, which could contribute to saving lives. Every small discovery could represent a big step in curing more people, such as by making more effective treatments available, diagnosing cancer earlier, or reducing the risk for cancer.

This Special Issue, “Advances in Cancer Therapy from Research to Clinical Practice—Surgical, Molecular or Systemic Management of Cancer”, was initially designed to allow the potential authors to share their work in the very complicated world of oncology using a large amount of big data, which can be practice changing.

The published articles covered a broad range of cancers, with the most important primary tumours being treated from a fundamental research or clinical practice point of view.

For breast cancer, for example, Lisencu et al. [5] tried to determine molecular predictive factors which are linked to the probability of obtaining a pathological response to neoadjuvant treatment. Selecting early those patients with the best chance of responding to a selective (and costly) therapy remains a challenging task. miRs could represent a good surrogate for the real-time evaluation of the tumour and its reaction to a specific treatment.

Moreover, Mkrantonakis et al. [6] tried to identify triple-negative breast cancer (TNBC) patients who have a better or worse response to immunomodulator agents, which are newcomers in the breast cancer armamentarium. PD-L1 gene polymorphisms rs822336 (G > C) and rs822337 (T > A) seem to be differentially expressed in TNBC patients and could represent potential markers of unfavourable prognosis and diminished survival. Ortega et al. [7] showed that the expression of insulin receptor substrate 4 (IRS-4), cyclin D1, and cyclooxygenase 2 (COX-2) are overexpressed in invasive lobular breast cancer compared to invasive ductal cancer, which could explain the difference between these two types of breast cancer in terms of prognosis and survival. 

The molecular types of cancer are not a fundamental discovery without any clinical relevance. As shown by Szep et al. [8], multiparametric breast MRI (magnetic resonance imaging), which provides morphological and functional images of breast tumours, is nowadays a part of the recommended workup for breast cancer. The molecular types can change the MRI aspect of breast cancer, whereas MRI can be used to predict the molecular types (but not replace the biopsy).

For lung cancer patients, Ahn et al. [9] searched for the expression of BRAF mutations in real-life unselected patients with non-small cell lung cancer. Despite reduced incidence in the analysed cohort, adenocarcinoma with micropapillary components seemed to have a privileged histology which harboured this mutation; this is an interesting finding that underlines the necessity of prioritising BRAF mutation testing.

In melanoma cancer, rechallenging with BRAF inhibitors becomes an attractive option for multiple-relapse patients. The polyclonal theory of cancer remains an important element to be taken into consideration when we are in a difficult situation after standard therapy failure. Ksomidis et al. [10] showed some interesting approaches to this clinical issue.

Inflammation in cancer could be a target for immunomodulatory treatment, but could also represent an unfavourable factor for patients, being responsible for cancer progression. The combined peripheral neutrophil–platelet index seems to be an unfavourable predictor factor for overall survival in resectable oesophageal squamous cell carcinoma (ESCC) patients, as shown by Peng et al. [11]. 

An absolute iron deficiency in colorectal cancer can impair not only the cardiac and respiratory functions, among others, but also the immune system’s defence. Deficient patients could be affected more and could present with more advanced disease–lymphatic invasion, as shown by Fagarasan et al. [12].

For hepatocarcinoma, Chen et al. [13] showed that recurrence with a surgery option can be alternatively treated with microwave ablation (MWA) that is either associated or not associated with transarterial chemoembolisation (TACE). Whether TACE adds something or not in terms of survival or diminishes the incidence of relapse remains a controversial issue. Most likely, the previous treatment could more appropriately select the best candidates. For those with progression of hepatocarcinoma after TACE, an option was proposed by Wu et al. [14] using the association of oxaliplatin with raltitrexed (HAICROX).

A new chemotherapy association or a new modality of drug delivery? Chioreanu et al. [15] tested a compound of 215 nm with an improved capacity of encapsulation, a good rate of diffusion of the drug, and a better tolerance potential.

Body mass index can be a simple prognostic factor for prostate cancer, as shown in Popovici et al.’s article [16], due to its relative resistance to insulin and possible proliferative stimulation, which could be responsible for a more advanced stage of disease at diagnosis.

Al-Gharaibeh et al. [17] analysed the tridimensional changes in the earliest cellular events before the start of renal cancer. In testicular cancer, Rohozneanu et al. [18] analysed if ‘less is more’ for stage I to avoid unnecessary toxicities.

For gynaecologic cancers, Obradovic et al. [19] investigated the role of a protein from the immunophilin family with antiangiogenic properties, which have a significantly decreased expression in the case of endometrioid endometrial carcinoma compared with benign endometrial hyperplasia. Havasi et al. [20] demonstrated the molecular mechanism of platin hypersensitivity and the strategies to overpass it, assuring the continuation of the essential regimen with platin derivates in ovarian cancer. As shown by Boeriu et al. [21], this class of medication could induce even a benign pathology such as lipomatosis in children.

Multiple neoplasia represents a difficult pathology for an oncologist, as mentioned and developed in an article published in *Medicina* by Simescu et al. [22]. Parathyroid carcinoma and differentiated thyroid carcinoma are exceptionally rare conditions. Less than 100 cases of adrenal neuroblastoma in adult patients have been reported, with one of these being described by Telecan et al [23]. A rare clinical condition represented by Maffucci syndrome and astrocytoma with a rare IDH mutation was described by Ashirov et al. [24].

Cotorogea-Simion et al. [25] developed the mechanisms presumed to be implicated in the pathogenesis of respiratory insufficiency, which frequently occurs in hematologic diseases. 

We hope that the readers will find answers to their questions or read about a finding related to their scientific interest in our Special Issue.
